# Holistic and component plant phenotyping using temporal image sequence

**DOI:** 10.1186/s13007-018-0303-x

**Published:** 2018-05-10

**Authors:** Sruti Das Choudhury, Srinidhi Bashyam, Yumou Qiu, Ashok Samal, Tala Awada

**Affiliations:** 1 School of Natural Resources, University of Nebraska-Lincoln, Lincoln, NE USA; 2 Department of Computer Science and Engineering, University of Nebraska-Lincoln, Lincoln, NE USA; 3 Department of Statistics, University of Nebraska-Lincoln, Lincoln, NE USA

**Keywords:** Plant phenotyping, Plant architecture, Holistic phenotypes, Component phenotypes, Image sequence analysis

## Abstract

**Background:**

Image-based plant phenotyping facilitates the extraction of traits noninvasively by analyzing large number of plants in a relatively short period of time. It has the potential to compute advanced phenotypes by considering the whole plant as a single object (holistic phenotypes) or as individual components, i.e., leaves and the stem (component phenotypes), to investigate the biophysical characteristics of the plants. The emergence timing, total number of leaves present at any point of time and the growth of individual leaves during vegetative stage life cycle of the maize plants are significant phenotypic expressions that best contribute to assess the plant vigor. However, image-based automated solution to this novel problem is yet to be explored.

**Results:**

A set of new holistic and component phenotypes are introduced in this paper. To compute the component phenotypes, it is essential to detect the individual leaves and the stem. Thus, the paper introduces a novel method to reliably detect the leaves and the stem of the maize plants by analyzing 2-dimensional visible light image sequences captured from the side using a graph based approach. The total number of leaves are counted and the length of each leaf is measured for all images in the sequence to monitor leaf growth. To evaluate the performance of the proposed algorithm, we introduce University of Nebraska–Lincoln Component Plant Phenotyping Dataset (UNL-CPPD) and provide ground truth to facilitate new algorithm development and uniform comparison. The temporal variation of the component phenotypes regulated by genotypes and environment (i.e., greenhouse) are experimentally demonstrated for the maize plants on UNL-CPPD. Statistical models are applied to analyze the greenhouse environment impact and demonstrate the genetic regulation of the temporal variation of the holistic phenotypes on the public dataset called Panicoid Phenomap-1.

**Conclusion:**

The central contribution of the paper is a novel computer vision based algorithm for automated detection of individual leaves and the stem to compute new component phenotypes along with a public release of a benchmark dataset, i.e., UNL-CPPD. Detailed experimental analyses are performed to demonstrate the temporal variation of the holistic and component phenotypes in maize regulated by environment and genetic variation with a discussion on their significance in the context of plant science.

## Background

The complex interaction between genotype and the environment determines the phenotypic characteristics of a plant which ultimately influences yield and resource acquisition. Image-based plant phenotyping refers to the proximal sensing and quantification of plant traits based on analyzing their images captured at regular intervals with precision. It facilitates the analysis of a large number of plants in a relatively short period of time with no or little manual intervention to compute diverse phenotypes. The process is generally non-destructive, allowing the same traits to be quantified repeatedly at multiple times during a plant’s life cycle. However, extracting meaningful numerical phenotypes based on image-based automated plant phenotyping remains a critical bottleneck in the effort to link intricate plant phenotypes to genetic expression.

**Fig. 1:**
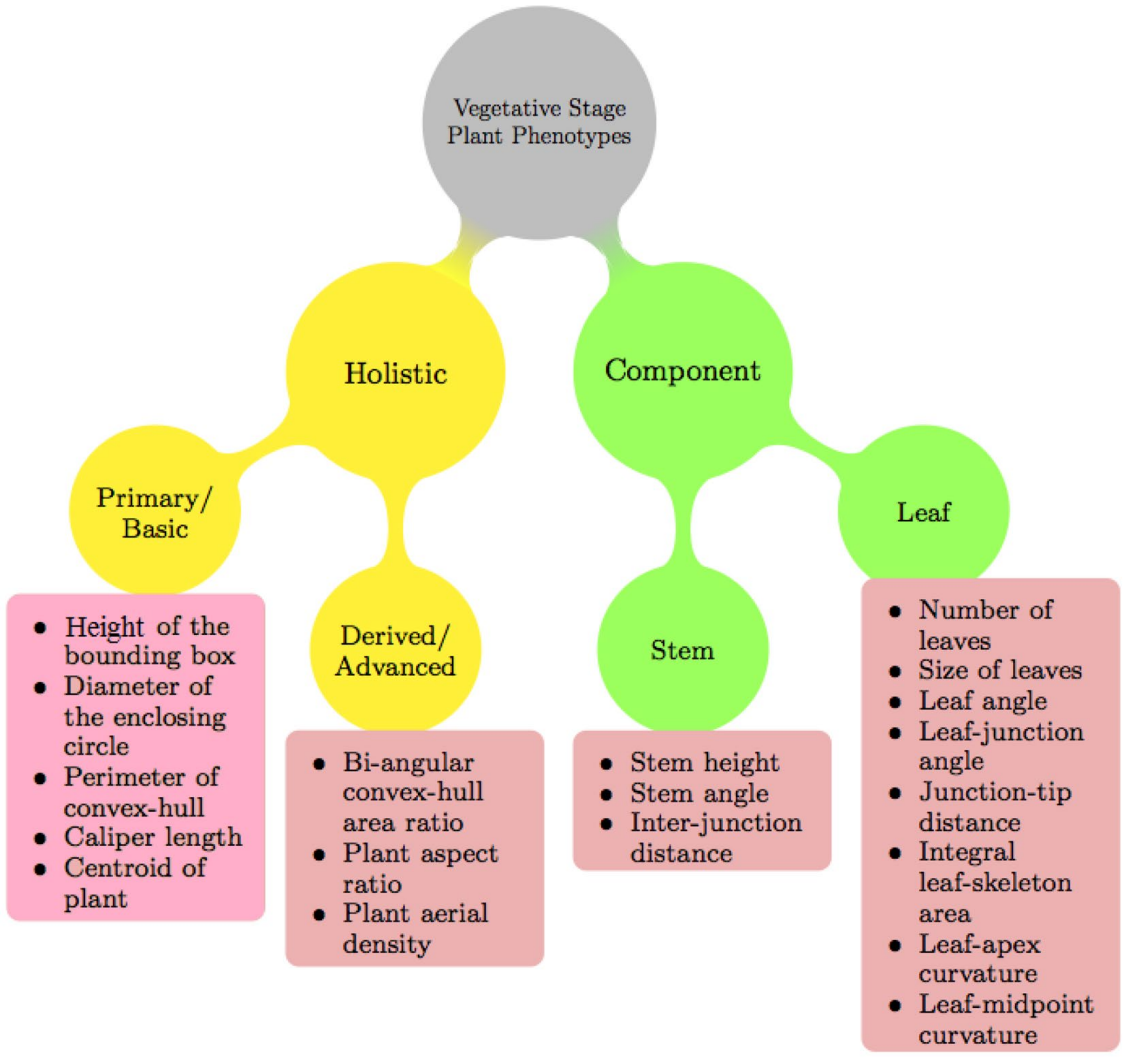
Categorization of vegetative stage plant phenotypes

The analysis of visible light (i.e., RGB) image sequence of plants for phenotyping is broadly classified into two categories: holistic and component-based [1]. Holistic analysis considers the whole plant as a single object and generates phenotypic values such as total pixel counts or metrics that quantify the basic geometric properties of the plant (e.g., height, width, plant aspect ratio, etc). Component-based analysis requires first identifying and distinguishing specific structures of a plant such as leaves, stem, or floral organs, and either quantifies properties of these structures individually or quantifies relationships between them. Figure 1 shows a high-level organization of vegetative stage image-based plant phenotypes. In contrast to component analysis, holistic analysis is simpler once proper segmentation of an image into plant and non-plant pixels has been performed. Therefore, most of the algorithms to compute plant phenotypes from images use holistic phenotypes. Holistic analysis is further divided into two categories, namely primary or basic and derived or advanced. Primary holistic phenotyping analysis measures the individual attributes of the basic geometrical shape, e.g., height of the bounding rectangle of a plant to quantify plant height, area of the convex-hull to quantify plant size. Derived holistic phenotypes combine two or more primary phenotypes for advanced plant phenotyping analysis. Component-based plant phenotyping analysis requires identifying and tracking individual plant structures that often have similar shape and appearance, which pose challenges. The development of effective component based plant phenotypes is important since they have the potential to improve our understanding of plant growth and development at a higher resolution.

Maize (*Zea mays*) or corn, has been the preeminent model for studying plant genetics over the past century, and is widely employed in both private and public sector research efforts in Asia, Europe, and the Americas. Maize is one of the three grass crops, along with rice and wheat, that directly or indirectly provides half of the total world caloric consumption each year. Arabidopsis and Tobacco have been widely used as the model plants for various applications in computer vision based plant phenotyping, i.e., leaf segmentation using 3-dimensional histogram cubes and superpixels [2], plant growth and chlorophyll fluorescence under various abiotic stress conditions [3], quantification of plant growth, photosynthesis, and leaf temperature-related parameters through the analysis of RGB, fluorescent light, and infrared time-lapse image sequences [4], automated plant segmentation using active contour model [5] and the rate of leaf growth monitoring based on leaf tracking using infrared stereo image sequences [6]. In contrast, extraction of phenotypes from the images of cereal crops, e.g., maize and sorghum, is only in the budding stage. The method in [1] introduces two derived holistic parameters namely bi-angular convex-hull area ratio and plant aspect ratio, which respectively contribute to the understanding of plant rotation due to shade avoidance and canopy architecture. This paper proposes an additional holistic phenotype called plant aerial density. While the method in [1] focuses on heritability analysis of the holistic phenotypes using boxplots, the proposed method applies statistical models to analyze the impact of a greenhouse environment and demonstrates the genetic regulation of the temporal variation of these phenotypes. Unlike the method in [1] which focuses on vegetative stage phenotyping analysis of maize, the method in [7] develops a robot-assisted imaging pipeline to track the growths of ear and silks based on an ear detection algorithm. The genotypic variation in silk growth rate under drought stress has been experimentally demonstrated. The method in [8] analyses the structure of a rice panicle based on image analysis, detects and counts the grains, and measures their shape parameters.

A 3D model of a sorghum (*Sorghum bicolor*) plant is reconstructed in [9] by using images acquired by a depth camera to identify quantitative trait loci for measuring shoot height, leaf angle, leaf length and shoot compactness. The method in [10] experimentally demonstrated the temporal variation of leaf angle and leaf area induced by light interception based on 3D reconstruction of maize plants from multiple side view images. The use of skeletonization process in the determination of plant architecture has been successfully demonstrated in [11, 12]. The method in [1] introduces a basic algorithm for leaf detection, where the leaf tips and leaf junctions are identified by inspecting the neighboring pixels of the skeleton of the leaf. A set of phenotypic traits, e.g., morphological, leaf architectural, textural and color-based, have been extracted from maize plants based on leaf and stem identification following skeletonization of binary images in [13]. The traits are used for yield prediction using QTL mapping that reveals genetic architecture of maize. However, for thin architectures like maize where complexity in shape and appearance increases over time, the skeletonization process often results in the formation of unwanted spurious branches. Since the methods in [1, 13] do not employ any technique to remove these spurious branches which are often falsely identified as leaves, the success is limited to early growth stages. This paper introduces an advanced algorithm for detecting individual components of a plant, i.e., leaves and the stem, using a robust graph based approach.

Graphical representations of skeletons have been investigated in the literature for many object recognition problems [14]. The method in [14] uses a skeletal graph to model a shape in order to use graph matching algorithms to determine similarity between objects. The method in [15] developed an ImageJ application to transform the skeleton of a shoot to a weighted graph, and uses the Floyd-Warshall shortest path algorithm to measure the shoot length of submerged aquatic plants. In this paper, we introduce the application of graphical representation of skeleton for detecting individual components of a plant, i.e., leaves and stem, to exploit the advantages of several concepts of graph theory. The skeleton of the plant is represented by a connected graph consisting of nodes and edges, where the nodes are labeled as either tips or junctions based on analyzing their degrees. A graph traversal algorithm is employed for efficient leaf detection. The length of each edge is analyzed to remove spurious branches based on thresholding. The weight associated with each edge represents the number of the leaf in order of emergence.

## Available phenotyping imaging tools and datasets

### Phenotyping imaging tools

Table 1 provides a comparative analysis of the existing shoot phenotyping software systems. The state-of-the-art image-analysis based plant phenotyping software systems are listed in http://www.plant-image-analysis.org/. This paper aims to introduce new component phenotypes by characterizing the leaves and stem of the plants that have not been considered in the literature. While the methods in [16–19] compute leaf area, leaf angle, indent width and height, we define a set of new phenotypes, e.g., junction-tip distance, leaf curvature, integral leaf-skeleton area, leaf-junction angle and stem angle, with a discussion on their significance in plant science. A use-assisted software tool called Leaf Extraction and Analysis Framework Graphical User Interface (Leaf GUI) is proposed in [20] for analyzing the structure of leaf venation networks and areoles. The methods in [21–24] computes primary holistic phenotypes, e.g., height and width of the plants, shoot area and biomass, whereas the proposed method computes three derived holistic phenotypes, e.g., bi-angular convex-hull area ratio, plant aspect ratio and aerial density, and demonstrate temporal variations of these phenotypes regulated by genotypes.

**Table 1:** State-of-the-art high throughput shoot phenotyping software tools

Software name	Plant part	Language	Target plant	Phenotype	Image type	Analysis technique	Comment
HTPheno [21]	Shoot	Java, ImageJ	Maize	x-extent, y-extent, diameter, width, height, projected shoot area	RGB	Open source, automated	Fails to handle changing light conditions and multiple zoom levels
Canopy reconstruction [22]	Shoot	C# using .NET	Rice, wheat	Shape and surface area of the leaf, shoot architecture (3D modeling)	RGB	Open source, automated	Multiple images captured using single camera from different angles. VisualSFM used to generate camera calibration and PMVS for point cloud generation in 3D reconstruction. Effected by occluded leaves or overlapping surfaces
Integrated analysis platform (IAP) [23]	Shoot	Java, ImageJ	Barley, maize, Sorghum	Morphological parameters, and watering status	RGB,F,NIR	Open source, semi automated	Supports cluster computing and can be expanded by the end user by implementing new algorithms
Rosette Tracker [4]	Leaf	Java, ImageJ	Rosette type (Arabidopsis)	Projected rosette area, maximal diameter, stockiness, compactness, growth rate, temperature	RGB, IR, F	Open source, semi automated	Only tested on rosette type plant (Arabidopsis)
PlantCV [18]	Shoot, leaf	OpenCV, Python, NumPy and MatPlotlib	Setaria	Height, width, convex-hull, biomass and leaf area	RGB, F, NIR	Open source, semi automated	Computes details of the plant at only holistic level and not at individual component level. Hardware used to capture image - LemnaTec Scanalyzer$^{3D-HT}$
Leaf shApe deterMINAtion (LAMINA) [16]	leaf	Java, ImageJ	All leaf types	Leaf shape, area, quantify leaf serration, missing leaf area, indent width, depth.	RGB	Open source	Automated and semi-automated platform-independent software tool under license GNU GPL2. First open source tool for for quantification of leaf serration. Results are affected when leafs are non symmetrical in shape
Black spot [17]	Leaf	Python	All leaf types	Leaf area	RGB	Free software	Batch leaf processing, leaf imaged under flatbed scanner
LEAF GUI [20]	Leaf	Matlab	All leaf types	Leaf extension analysis-area state, vein state, areole state	RGB	Open source, semi automated	Destructive analysis
Circumnutation tracker [24]	Shoot	C++	Helianthus annuus	Circumnutation parameters: period, length, rate, shape, and clockwise- and counter-clockwise directions	Black/white or color time-lapse video images	Open source	Only tested on sunflower (*Helianthus annuus*)
Panicle TRAit phenotyping (P-Trap) [8]	Fruit	JAVA on Netbeans 7.3	Rice	Architecture of rice panicle, shape of seed, grain counting and detection	RGB	Opensource	Skeletonization process cannot accurately deal with curved panicle axes, and mislabel hair-like extensions on rice spikelets as branches
Leaf angle distribution toolbox [19]	Leaf	Matlab	Sugar beet	Leaf surface and leaf angle	RGB	Open source, semi automated	Requires stereo camera setup. Manual intervention for leaf segmentation for dense canopies

Keys-*RGB* red–green–blue, *IR* infrared, *NIR* near infrared, *F* fluorescent

### Existing datasets

The publicly available datasets for computer vision based plant phenotyping have mainly considered Arabidopsis (*Arabidopsis thaliana*) and tobacco (*Nicotiana tabacum*) [25, 26]. The leaf segmentation challenge (LSC) dataset [25] consists of three subsets: A1 (Ara2012), A2 (Ara2013) and A3(Tobacco). Ara2012 and Ara2013 subsets consist of top-view time-lapse images of *Arabidopsis thaliana* rosettes. The total number of images in Ara2012 and Ara2013 are 150 and 5048, respectively. A3 dataset consists of top-view images of tobacco (*Nicotiana tabacum*) plants which are captured hourly by a robot equipped with two stereo camera systems for 30 days. The LSC dataset is publicly available from http://www.plant-phenotyping.org/CVPPP2014-challenge.

The Plant Imagery Dataset developed at the Michigan State University (MSU-PID dataset) [26] consists of images of Arabidopsis (total 2160 $\times$ 4 images) and bean (total 325 $\times$ 4 images) captured by 4 types of calibrated cameras, i.e., fluorescent, IR, RGB color and depth sensor to facilitate phenotyping research in the areas of leaf segmentation, leaf counting, leaf alignment, leaf tracking and 3D leaf reconstruction. A subset (576 $\times$ 4 Arabidopsis images and 175 $\times$ 2 bean images) is annotated to provide ground truth for leaf tip location, leaf segmentation and leaf alignment. The dataset consists of images of a single genotype, and hence not suitable for research on genetic regulation of phenotypes. MSU-PID dataset is publicly available from http://cvlab.cse.msu.edu/multi-modality-imagery-database-msu-pid.html [26].

To stimulate plant phenotyping research in the case of panicoid grain crops, a public dataset called Panicoid Phenomap-1 is introduced in [1]. It consists of visible light image sequences of 40 genotypes including at least one representative accession from five panicoid grain crops: maize, sorghum, pearl millet, proso millet, and foxtail millet. The dataset does not contain any ground truth, as it is primarily designed for the development and evaluation of holistic phenotypes. However, to evaluate the performance of the leaf and stem detection algorithm and validate the correctness of the component phenotypes, a benchmark dataset with human-annotated ground truth is indispensable. Since such a dataset is not publicly available, we introduce the University of Nebraska–Lincoln Component Plant Phenotyping Dataset (UNL-CPPD) to spur research in leaf detection and tracking, leaf segmentation, evaluation of holistic and component-based phenotypes, and identifying new research problems in computer vision based phenotyping analysis of the maize plants and also other cereal crops sharing similar architecture, e.g., sorghum.

## Methods

The proposed method has three phases: (a) view selection; (b) determination of plant architecture using a graph based approach; and (c) computation of holistic and component phenotypes.

### View selection

**Fig. 2:**
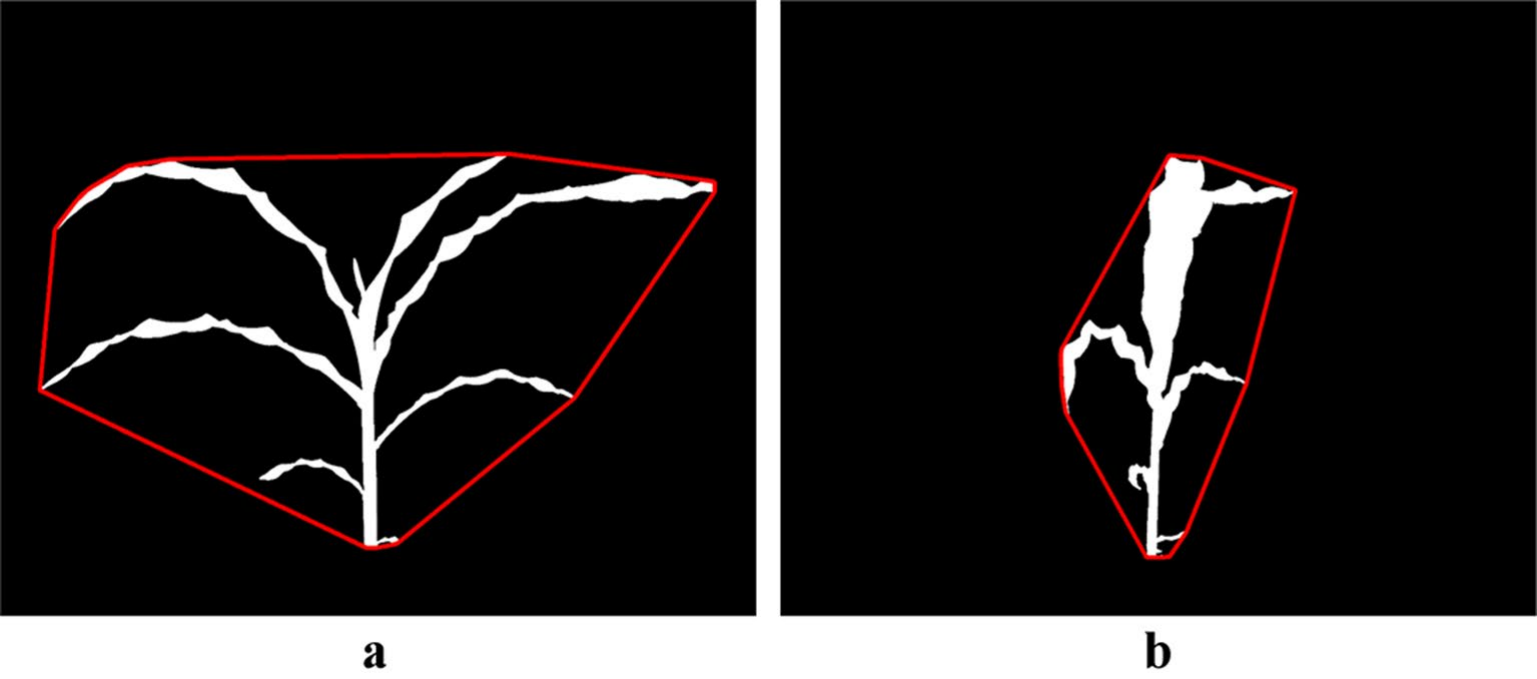
Illustration of view selection: **a** binary image of a maize plant enclosed by convex-hull at side view 0$^\circ$; and **b** binary image of the same maize plant enclosed by convex-hull at side view 90$^\circ$

View selection is a prerequisite for the plant architecture determination algorithm. A plant image sequence consists of images captured on increasing days from multiple view angles. To achieve maximum efficiency, it is best to analyze the plant images captured from that particular view angle at which the line of sight of the camera is perpendicular to the line of axis of the leaves. To ensure the automatic selection of the view at which the line of sight of the camera is perpendicular to the line of axis of the leaves, we compute the area of the convex-hulls of the plant images of all available views for each day. For each imaging day, the view at which the area of the convex-hull of the plant is the maximum, is selected for subsequent analysis.

For simplicity and without loss of generality, we consider two side views, i.e., side view 0$^\circ$ and side view 90$^\circ$. The view selection process is summarized in Eq. 1.

1 $$\begin{aligned} \alpha _p=\left\{ \begin{array}{ll} 0^\circ \quad \text {if} &{}\quad (\text {CV-area0}_p)> (\text {CV-area90}_p) \\ 90^\circ \quad \text {if} &{}\quad (\text {CV-area90}_p) > (\text {CV-area0}_p)\\ \end{array} \right. \end{aligned}$$

where $\text {CV-area0}_p$ and $\text {CV-area90}_p$ denote the area of the convex-hulls of the images on the *p*-th day at side view 0$^\circ$ and side view 90$^\circ$, respectively. If the area of the convex-hull at side view 0$^\circ$ is higher than the area of the convex-hull at side view 90$^\circ$, the image of side view 0$^\circ$ is chosen for that day for subsequent analysis and vice-versa. Figure 2a, b show the binary images of a maize plant from Panicoid Phenomap-1 captured from two different side views, i.e., 0$^\circ$ and 90$^\circ$, respectively, and their convex-hulls (shown in red). It is readily apparent from the figures that the area of the convex-hull at side view 0$^\circ$ is higher than the area of the convex-hull at side view 90$^\circ$.

### Plant architecture determination

In this section we describe the process of plant architecture determination, a full outline can be found in Algorithm 1.

#### Segmentation

**Fig. 3:**
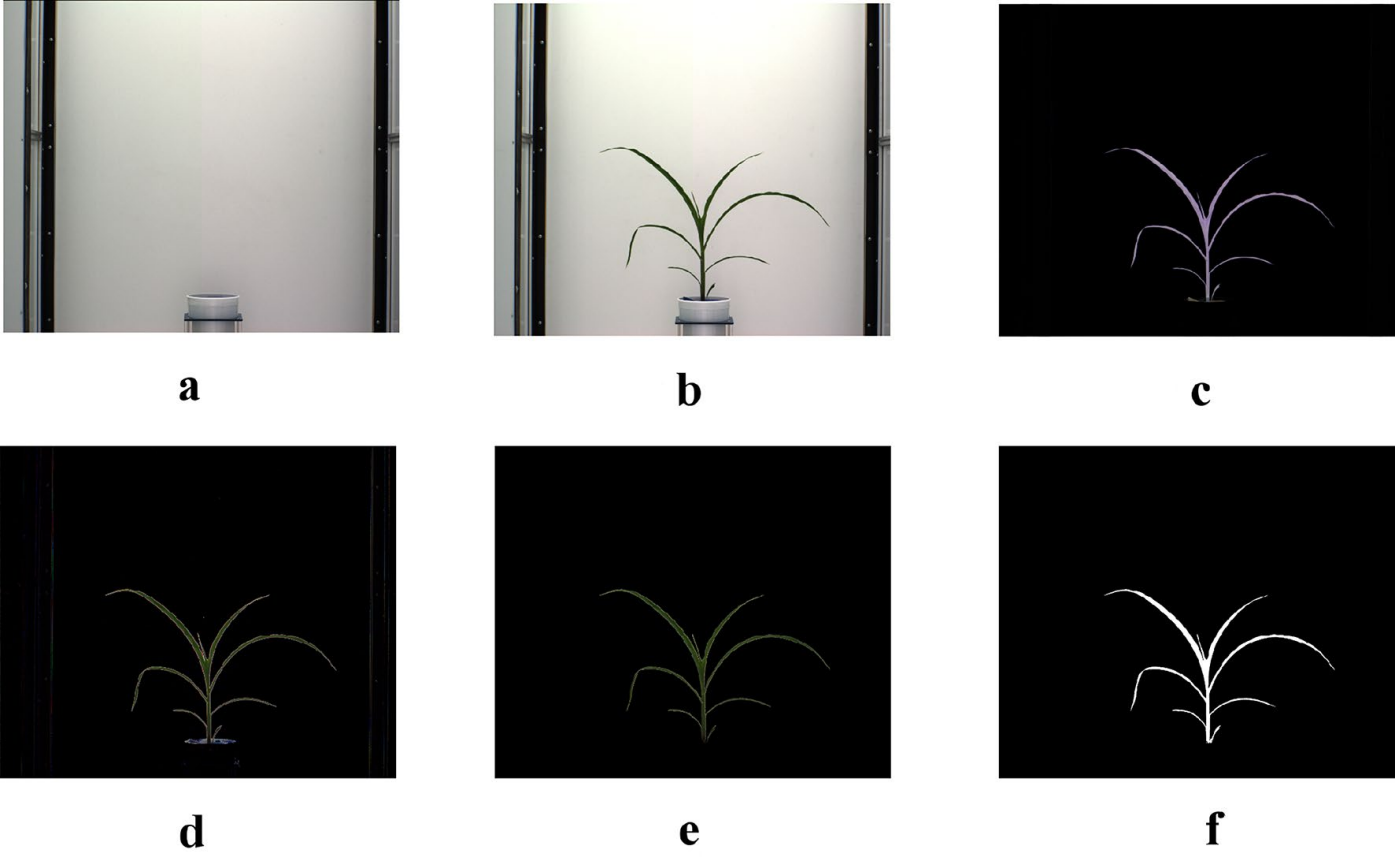
Illustration of segmentation process: **a** background image; **b** original image; **c** foreground obtained after applying frame differencing technique; **d** foreground obtained by green pixel superimposition; **e** foreground containing green pixels characterizing the plant; and **f** binary image

The first step is to segment the plant (foreground), from the background, i.e., the part of the scene which remains static over the period of interest for the image sequence. Since, the imaging chambers of Lemnatec Scanalyzer 3D high throughput plant phenotyping system has a fixed homogeneous background, the simplest background subtraction technique based on frame differencing is used to extract the foreground. However, successful execution of this technique requires the background and foreground images to be aligned with respect to scale and rotation. Hence, prior to applying frame differencing technique of background subtraction, we used automated image registration technique based on local feature detection and matching to account for change in zoom levels (resulting in scale variation) during the image capturing process. The key to feature detection is to find features (e.g., corners, blobs and edges) that remain locally invariant so that they are detected even in the presence of rotation and scale change [27]. In the proposed method, the corners of the pots, the pot center and the edges of the frame of the imaging cabinet are used as the local features for aligning the foreground and the background based on correspondence detection. Figure 3a, b respectively show the background and the original image. The extracted foreground as shown in Fig. 3c resulting from frame differencing technique of background subtraction, retains some pixels of the background due to lighting variations. It also retains undesirable part of the plant, e.g., soil, soil covering film, etc.

In order to remove resulting noises due to variation in lighting, the green pixels of the original image are superimposed onto Fig. 3c, which results in the image as shown in Fig. 3d. The green pixels constituting the plant are retained, while nosy pixels of other colors are set to zero values to make them part of the background. Thus, the noises are removed. The resulting foreground consisting of only green pixels characterizing the plant is shown in Fig. 3e. A color-based thresholding in HSV (Hue, Saturation and Value) color space is applied on this image using the following ranges: hue (range 0.051–0.503), saturation (range: 0.102-0.804) and value (range 0.000–0.786) to binarize the image. The resulting binary image is shown in Fig. 3f. The binary image is subjected to connected-component analysis involving morphological operation of erosion to remove noisy pixels and followed by dilation to fill up any small holes inside the plant image to give a single connected region as shown in Fig. 3f.

#### Skeletonization

**Fig. 4:**
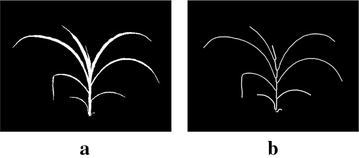
Illustration of skeletonization: **a** binary image; **b** skeleton image

**Fig. 5:**
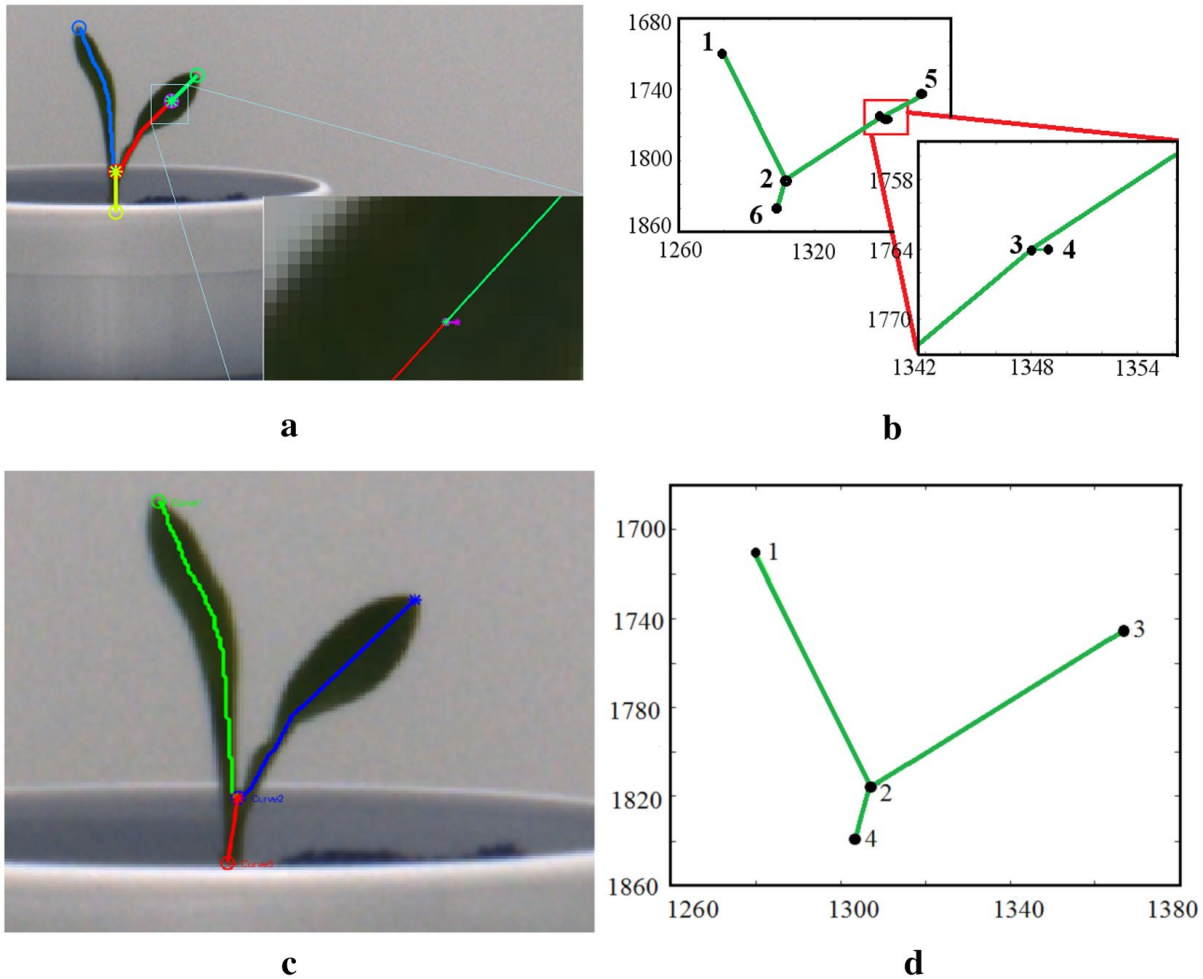
Illustration of spur removal process: **a** Spurious branch giving rise to a false node in the leaf; **b** visualization of Spur in the graphical representation of the plant; **c**, **d** Spur removal based on threshold based skeleton pruning in the original plant and its graphical representation

The skeletonization, i.e., the process of reducing a shape to one-pixel wide lines that preserve the shape’s main topological and size characteristics, are mainly computed based on morphological thinning, geometric methods and fast marching distance transform. The morphological thinning based methods iteratively peel off the boundary layer by layer, identifying the points whose removal does not affect the shape topology. Although straightforward to implement, it requires intensive heuristics to ensure the skeletal connectivity, and hence does not perform well in the case of complex dynamic structures like plants. The geometric methods compute Voronoi diagram to produce accurate connected skeleton. However, its performance largely depends on the robustness of the boundary discretization, and is computationally expensive. We used fast marching distance transform to skeletonize the binary image as explained in [28] due to its robustness to noisy boundaries, low computational complexity and accuracy in terms of skeleton connectivity. Figure 4a shows the binary image of a plant and Fig. 4b shows the corresponding skeleton image.

The limiting factor of skeletonization process is the skeleton’s high sensitivity to boundary noises generating redundant spurious branches or spurs, which significantly affects the topology of the skeleton graph [29]. The most common approaches to overcome skeleton instability are based on skeleton pruning, i.e., eliminating redundant skeleton branches. Figure 5a, b respectively show the spurious branches resulting from the skeletonization process in the original plant and its corresponding graphical representation. We use thresholding based skeleton pruning to remove spurious branches, i.e., if the length of an edge is $\le$ threshold, is it regarded as a spur, and hence discarded. The value of threshold is chosen as 10 pixels for our method. It is observed that all spurs are removed using this threshold value for all plant image sequences of UNL-CPPD. The process of skeleton pruning, i.e., the elimination of spurious branches from the skeleton [29], leaves redundant degree-2 nodes from which the spurious branches originated. These redundant nodes are also removed so that a leaf or an inter-junction is represented by a single edge. Figure 5c, d respectively show that the spur is removed in the original plant image and its graphical representation based on the skeleton pruning process described above.

#### Graphical representation of plant

**Fig. 6:**
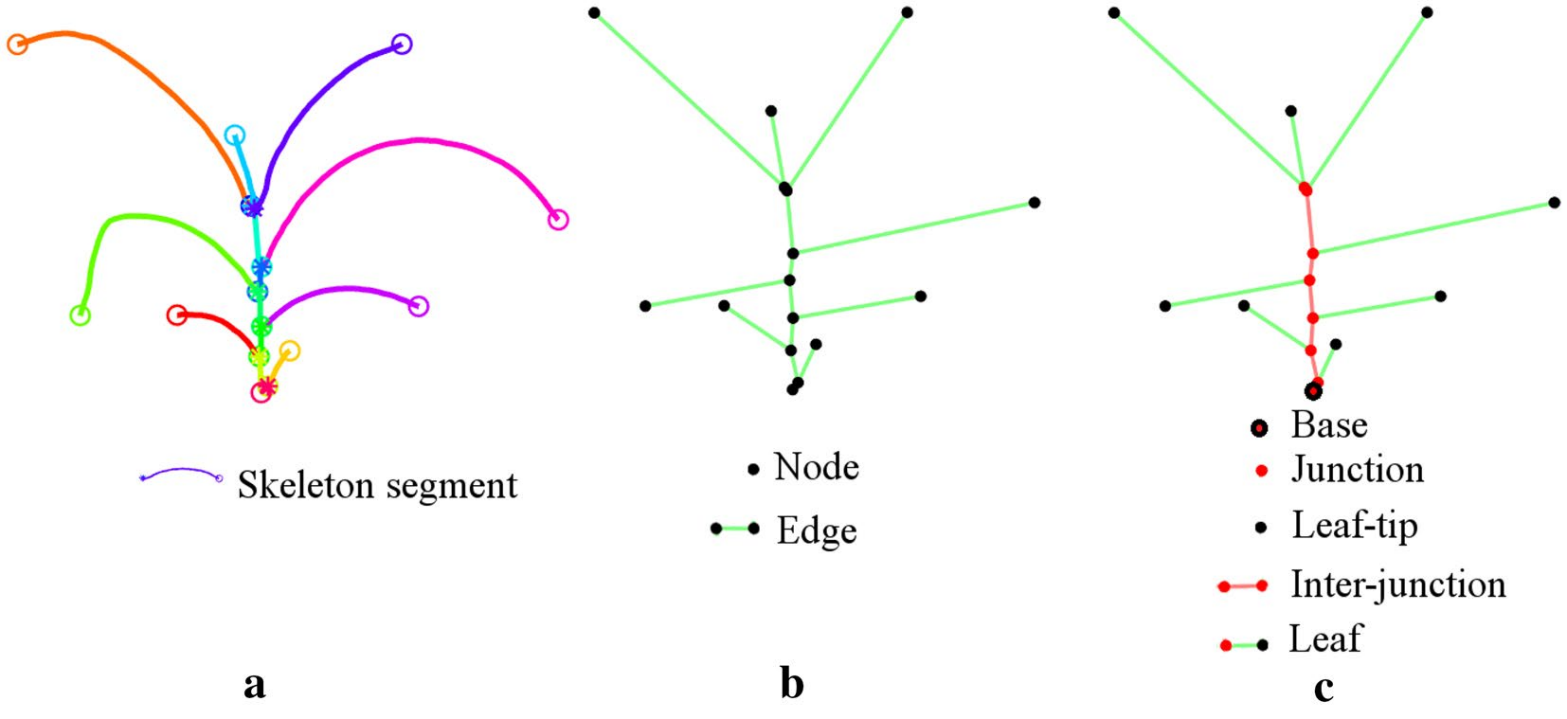
Plant architecture determination: **a** plant skeleton with each leaf marked with different colors; **b** graphical representation of the plant with nodes and edges; and **c** plant body-part labeling

The skeleton of the plant *P* is represented as the graph, i.e., *P* = {*V*, *E*}, where *V* is the set of nodes, and *E* is the set of edges. The set of nodes, *V*, are defined by *V* = {*T*, *B*, *J*} where *B*, *T*, and *J* are the base of the plant, the tips of leaves and the junctions in stem from which the leaves emerge, respectively. The set of edges *E* is defined as *E* = {*L*, *I*}, where, *L* and *I* represent the leaves and inter-junctions in the plant, respectively. These terms are briefly described below and are graphically shown in Fig. 6. Base (*B*): The base of the plant is the point from where the stem of the plant emerges from the ground and is the bottom most point of the skeleton. Junction (*J*): The node where a leaf is connected to the stem. This is also referred to as ‘collar’ in plant science. The junctions are nodes of degree 3 or more in the graph. Tip (*T*): The node with degree 1 is considered as a tip. it is the free end of the leaf. Leaf (*L*): Leaves connect the leaf tips and junctions on the stem. If an edge has one node that is a leaf tip, it is considered as a leaf. Inter-junction (*I*): The edge connecting two junctions are called inter-junctions. The stem is formed by iteratively traversing the graph from the base along a connected path of junctions.

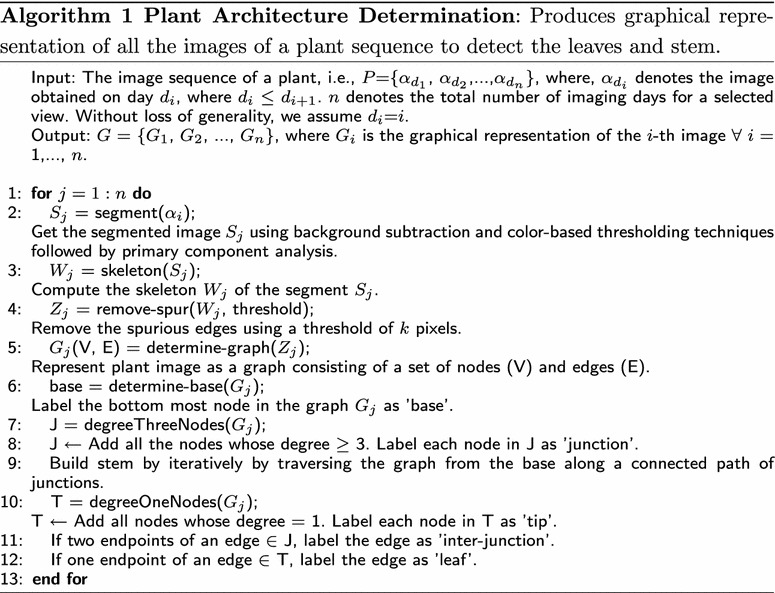



### Phenotype computation

#### Holistic phenotypes

The method presented in [1] introduced two holistic phenotypes, namely, bi-angular convex-hull area ratio $(BA_{CH}R)$ and plant aspect ratio (*PAR*), defined as follows:

2 $$\begin{aligned} BA_{CH}R=\frac{Area_{CH} \text { at side view } 0^{\circ }}{Area_{CH} \text { at side view 90}^{\circ }}, \end{aligned}$$

and

3 $$\begin{aligned} PAR = \frac{ Height_{BR} \text { at side view}}{Diameter_{MEC} \text { at top view}}, \end{aligned}$$

where, $Area_{CH}$ is the area of the convex-hull, $Height_{BR}$ denotes the height of the bounding rectangle (BR) of the plant in side view 0$^{\circ }$ and $Diameter_{MEC}$ denotes the diameter of the minimum enclosing circle (MEC) of the plant in top view.

The plant aerial density *PAD* is defined as

4 $$\begin{aligned} PAD = \frac{Plant_{Tpx}\text { at side view }0^{\circ }( 90^{\circ })}{Area_{CH} \text { at side view 0}^{\circ } (90^{\circ })}, \end{aligned}$$

where, $Plant_{Tpx}$ denotes the total number of plant pixels.

$BA_{CH}R$ provides information on plant rotation due to shade avoidance, whereas *PAR* is a measure which helps to distinguish between genotypes with narrow versus wide leaf extent when plant height is controlled. All these three holistic phenotypes are the ratios of two parameters with same units, and hence, they are scale invariant.

#### Component phenotypes

**Fig. 7:**
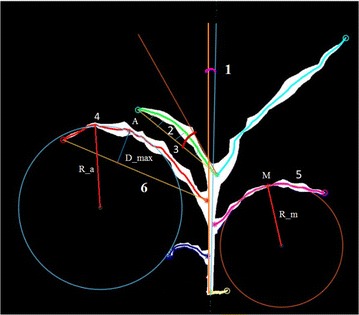
Component phenotypes: 1-stem angle; 2-integral leaf-skeleton area; 3-leaf-junction angle; 4-apex curvature; 5-mid-leaf curvature; and 6-junction-tip distance

Here, we employ component-based approaches to quantify two aboveground, vegetative stage organs of a maize plant: the leaf and the stem (where the stem actually consists of stem tissue and multiple wrapped leaf sheaths). The image-based approaches employed here enable the quantification of a number of phenotypes currently scored by plant biologists using manual techniques such as total number of leaves, inter-junction distance, stem height, leaf length, and leaf angle. Furthermore, computer vision based phenotyping analysis also made it possible to measure a number of additional component-based phenotypes that would not be practical to measure using manual techniques such as (a) junction-tip distance; (b) integral leaf-skeleton area; (c) leaf-junction angle; (d) leaf curvature; and (e) stem angle. These component phenotypes are shown in Fig. 7.

*Leaf length:* It measures the length of each leaf. Let the *n*-th order polynomial equation generated by polynomial curve fitting of each leaf is given by

5 $$\begin{aligned} y=p(x)=p_1x^n+p_2x^{n-1}+p_3x^{n-2}+\cdots +P_nx+P_{n+1}, \end{aligned}$$

where, $p_1$, $p_1$,..., $p_{n+1}$ are the coefficients of the best fit polynomial for the leaf skeleton optimizing the least square error. The leaf length is measured using

6 $$\begin{aligned} \int _{x_1}^{x_2}\sqrt{1+(dy/dx)^2}, \end{aligned}$$

where, $x_1$ and $x_2$ denote the *x*-co-ordinates of the leaf-junction and leaf-tip, respectively.

*Junction-tip distance:* It is defined as a distance between the junction and the tip of each leaf measured using a straight line. Junction-tip distance is measured using

7 $$\begin{aligned} X = \sqrt{(x_2-x_1)^2+(y_2-y_1)^2}. \end{aligned}$$

where, ($x_1$,$y_1$) and ($x_2$,$y_2$) respectively denote the co-ordinates of the junction and tip of the leaf. The straight line connecting the tip and junction is called junction-tip path.

*Leaf curvature:* The steps to compute leaf curvature are given below. The *n*th order polynomial equation of the leaf skeleton is computed using Eq. 5. The radius of curvature (*R*) at any point on the leaf skeleton is given by

8 $$\begin{aligned} R = \frac{[1+(dy/dx)^2]^{\frac{3}{2}}}{|\frac{d^2y}{dx^2}|}. \end{aligned}$$

Hence, curvature (*K*) is given by

9 $$\begin{aligned} K = \frac{1}{R}. \end{aligned}$$

For a given radius of curvature at a specific point on the tangent of a curve, we get two centers for positive and negative values of x-coordinate. We consider the two neighboring points of the mid-leaf and join the points using a straight line. The circle of which this straight line is a chord, is considered.

We compute leaf curvature at two special points on the leaf skeleton, i.e., leaf apex and mid-leaf. Leaf apex is defined as the pixel at leaf skeleton, perpendicular distance of which is maximum from the junction-tip path. Mid-leaf is the mid-point of the leaf skeleton. Leaf curvature is divided into two types based on the point at which it is computed, i.e., (a) apex curvature and (b) mid-leaf curvature.

To compute leaf apex, we measure the perpendicular distance from all the points of the skeleton of the leaf and the junction-tip path using

10 $$\begin{aligned} dist = \frac{(y_2-y_1)x_0-(x_2-x_1)y_0+x_2y_1-y_2x_1}{\sqrt{(y_2-y_1)^2+(x_2-x_1)^2}}, \;\;\forall (x_0,y_0) \in {S}, \end{aligned}$$

where, *S* is the set of all points of the leaf skeleton. Then, we compute max(*dist*). Leaf apex is the point at which *dist* is the maximum. Note that there might be more than one leaf apex.

We compute mid-leaf as follows. Let *n* be the total number of points in the leaf skeleton. The mid-point *b* is computed as

11 $$\begin{aligned} \hbox {b} = floor (\hbox {n}/2). \end{aligned}$$

Mid-leaf is the point on *p*(*x*) which is at the x-intercept of *b*.

*Leaf-junction angle:* Leaf-junction angle, $\theta$, is defined as the angle between the tangent of the leaf at its point of contact with the junction and the junction-tip path. It is measured using

12 $$\begin{aligned} \theta = tan^{-1} \frac{m_2-m_1}{1+m_1m_2}, \end{aligned}$$

where, $m_1$ and $m_2$ respectively denote the slopes of the tangent to the leaf at its point of contact with the junction and the junction-tip path.

*Integral leaf-skeleton area:* It is defined as the area enclosed by the leaf and the straight line joining junction and tip of the leaf, i.e., junction-tip path. Let *p*(*x*) be the equation of the leaf computed by polynomial curve fitting. Let *f*(*x*) be the equation of the Junction-tip path which is computed by

13 $$\begin{aligned} f(x) = \frac{y_1-y_2}{x_1-x_2}(x-x_1)+y_1, \end{aligned}$$

where, ($x_1, x_2$) and ($y_1, y_2$) respectively denote the co-ordinates of the leaf-junction and leaf-tip. The leaf area enclosed by *p*(*x*) and *f*(*x*) is computed by

14 $$\begin{aligned} \int _{a}^{b}[p(x)-f(x)], \end{aligned}$$

where, *a* and *b* denote the x-co-ordinate of the leaf-tip and leaf-junction, respectively.

*Stem angle:* We define stem axis as the straight line formed by linear regression curve fitting of all the junctions of a stem. The stem angle ($\phi$) is defined as the angle between the stem axis and the vertical axis using

15 $$\begin{aligned} \phi =tan^{-1}(m), \end{aligned}$$

where, *m* is the slope of stem axis.

### Discussion on phenotypic significance

The plant vigor can be best interpreted by the growth of individual leaves over time, and thus, leaf length and junction-tip distance are the two important phenotypes. They also help in the study of determining plants’ response to environmental stresses. Leaf curvature is a measurement of toughness of a leaf. Leaf toughness appears to be an important defense mechanism in maize across diverse groups of germplasm. Computer vision based leaf curvature measurement will replace the manual and tedious process of using mechanical devices, e.g., penetrometres, to measure leaf toughness used in resistance breeding programs and studying phytochemical characteristics of leaves [30]. Stem angle, which is a measurement of deviation of stem axis from the vertical line, can be an early signal to lodging susceptibility. Yield loss due to lodging reduces the US corn harvest by 5–25% year (2.4–12 billion dollars at 2015 corn prices). Lodging is also an issue for farmers growing other grain crops including wheat, sorghum, and millet. The ratio of integral leaf-skeleton area to the junction-tip path provides information on leaf drooping, which could be an indicator of plant vigor such as nutrient deficiency.

## Dataset

This section provides discussion on two publicly available datasets, i.e., Panicoid Phenomap-1 and UNL-CPPD, respectively used for experimental analysis of holistic phenotypes and component phenotypes.

### Imaging setup

**Fig. 8:**
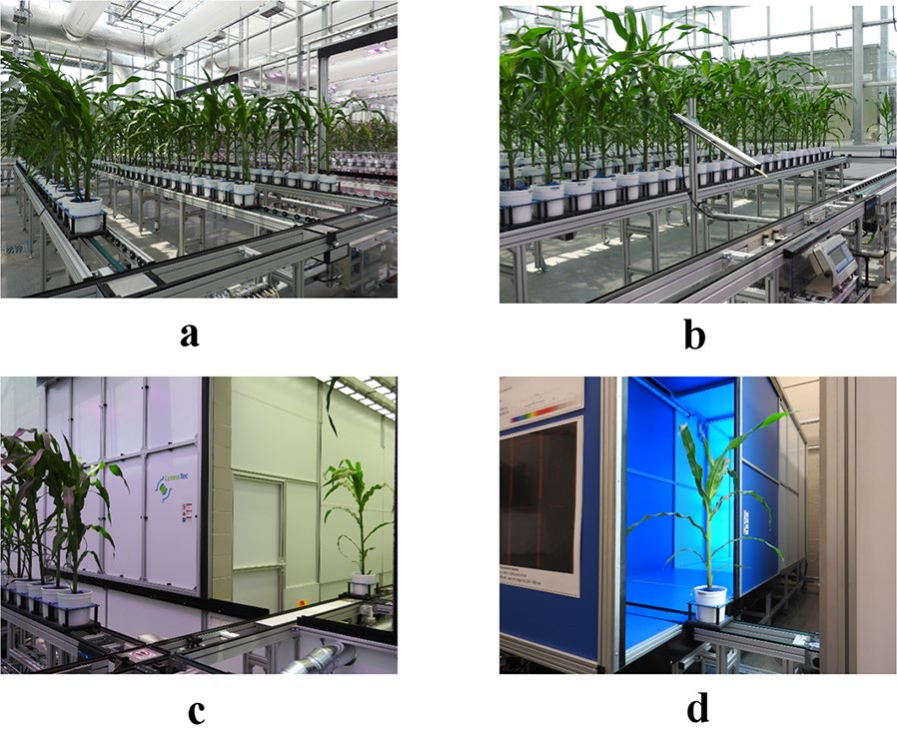
Lemnatec Scanalyzer 3D plant phenotyping facility at the UNL, USA, for high throughput plant phenotyping: **a** view of the greenhouse; **b** view of the greenhouse with watering station; **c** Lemnatec imaging chambers; and **d** plant entering into the fluorescent chamber

The University of Nebraska–Lincoln (UNL), USA, is equipped with the Lemnatec Scanalyzer 3D high throughput plant phenotyping system. Each plant is placed in a metallic carrier (dimension: 236 mm $\times$ 236 mm $\times$ 142 mm) on a conveyor belt that moves the plants from the greenhouse to the four imaging chambers successively for capturing images in different modalities. Table 2 shows the types and specifications for the different types of cameras. Each imaging chamber has a rotating lifter for up to 360 side view images. The conveyor belt can accommodate up to 672 plants with height up to 2.5 m. It has three watering stations with balance that can add water to target weight or specific volume, and records the specific quantity of water added on a daily basis. Figure 8a shows the view of the greenhouse equipped with the Lemnatec Scanalyzer 3D high throughput plant phenotyping system used for this research; Fig. 8b shows a watering station; Fig. 8c shows the imaging chambers; and Fig. 8d shows a plant entering into the fluorescent imaging chamber.

**Table 2:** Specifications of different types of cameras of the Lemnatec Scanalyzer 3D high throughput plant phenotyping system at the UNL, USA

Camera type	Spatial resolution (px)	Spectral range (nm)	Band	Frame rate (fps)	Bit depth (bit)
Visible light	$2454\,\times \,2056$	400–700	–	17	24
Fluorescent	$1390\,\times \,1038$	620–900	–	24	14
Infrared	$640\,\times \,480$	8–14	–	5	14
Near-infrared	$640\,\times \,480$	900–1700	–	24	14
hyperspectral	320 line width	545–1700	243	100	16

### Dataset organization

**Table 3:** The names of the genotypes corresponding to the genotype IDs used in the Panicoid Phenomap-1 dataset

$G_{ID}$	$G_{name}$	$G_{ID}$	$G_{name}$	$G_{ID}$	$G_{name}$	$G_{ID}$	$G_{name}$	$G_{ID}$	$G_{name}$
1	740	9	C103	17	LH82	25	PHG83	33	Yugu1
2	2369	10	CM105	18	Mo17	26	PHJ40	34	PI614815
3	A619	11	LH123HT	19	DKPB80	27	PHH82	35	PI583800
4	A632	12	LH145	20	PH207	28	PHV63	36	Purple Majesty
5	A634	13	LH162	21	DHB47	29	PHW52	37	BTx623
6	B14	14	LH195	22	PHG35	30	PHZ51	38	PI535796
7	B37	15	LH198	23	PHG39	31	W117HT	39	PI463255
8	B73	16	LH74	24	PHG47	32	Wf9	40	PI578074

**Table 4:** The experimental design for maize (ID: 1–32) and non-maize plants (ID: 33–40)

39	36	37	33	39	40	–	–	–	–
38	35	35	34	34	33	–	–	–	–
40	34	38	39	36	35	–	-	–	–
37	33	36	40	37	38	–	–	–	–
***20***	**12**	6	24	20	2	2	13	22	19
***18***	**8**	14	20	31	1	19	26	24	17
***4***	**28**	19	4	23	26	15	12	8	20
***2***	**15**	22	27	4	10	31	28	6	3
***21***	**30**	5	26	7	30	11	29	25	4
***29***	**14**	3	8	22	18	3	6	9	28
***5***	**31**	30	11	6	14	18	10	18	1
***13***	**24**	21	10	15	17	27	22	2	12
19	*22*	9	18	11	8	24	20	26	30
26	*6*	25	2	5	3	7	14	16	11
25	*27*	17	28	12	13	5	32	21	7
23	*17*	1	7	28	16	21	16	31	27
10	*32*	13	16	27	24	23	9	32	14
3	*1*	15	32	21	29	17	4	5	23
7	*16*	31	23	9	32	1	30	10	13
9	*11*	29	12	25	19	8	25	15	29

Different emphasis represent the examples of blocks used in the maize design. The genotype names corresponding to the genotype IDs are provided in Table  3

We introduced Panicoid Phenomap-1 dataset in [1]. The dataset consists of images of the 40 genotypes of panicoid grain crops including at least one representative accession from each of the five categories: maize, sorghum, pearl millet, proso millet and foxtail millet. The images were captured daily by the visible light camera for two side view angles, i.e., 0$^\circ$ and 90$^\circ$, for 27 consecutive days. Panicoid Phenomap-1 contains 13,728 total number of images from 176 plants. Table 3 shows the genotype names corresponding to genotype IDs used in the dataset. The imaging started on October 10, 2015, 2 days after planting the seeds. The dataset is designed to facilitate the development of new computer vision algorithms for the extraction of holistic phenotypic parameters specifically from maize and to encourage researchers to test the accuracy of these algorithms for related crop species with similar plant architectures.

We created a subset of Panicoid Phenomap-1 dataset consisting of images of the 13 maize plants to evaluate our component phenotyping algorithm. We call this dataset as UNL-CPPD. While Panicoid Phenomap-1 only contains original images captured by the visible light camera, UNL-CPPD is released with human-annotated ground truth along with the original image sequences to facilitate image-based component phenotyping analysis. The dataset will also stimulate research in the development and comparison of algorithms for leaf detection and tracking, leaf segmentation and leaf alignment of maize plants. The dataset will also motivate the exploration of components phenotypes and investigate their temporal variation regulated by genotypes.

The images of UNL-CPPD are captured by the visible light camera (BASLER: piA2400-17gc) in the Lemnatec Scanalyzer 3D high throughput plant phenotyping facility located at the innovation campus of the UNL, USA, once daily for 32 days. UNL-CPPD has two versions: UNL-CPPD-I (small) and UNL-CPPD-II (large). UNL-CPPD-I comprises images for two side views: 0$^\circ$ and 90$^\circ$ of 13 maize plants for the first 27 days starting from germination that merely exclude self-occlusions due to crossovers. UNL-CPPD-II comprises images for two side views: 0$^\circ$ and 90$^\circ$ of the same 13 plants for longer duration, i.e., 32 days to evaluate the proposed method in presence of leaf crossovers and self-occlusions. It should be noted that $Plant\_{104}-24$ has images for 31 days (Day 32 is unavailable) and $Plant\_{191}-28$ has images for 30 days (Day 27 and Day 32 are unavailable). Thus, UNL-CPPD-I contains total number of 700 original images and UNL-CPPD-II contains total number of 816 original images including the images contained in UNL-CPPD-I. Corresponding to each original image, the dataset also contains the ground truth and annotated image with each leaf numbered in order of emergence. We release the following ground truth information in the XML format for each original image of the plant: (a) the co-ordinates of leaf-tips and leaf-junctions; and (b) the total number leaves present (which are numbered in order of emergence). Both the datasets, i.e., Panicoid Phenomap-1 and UNL-CPPD can be freely downloaded from http://plantvision.unl.edu/. The sizes of Panicoid Phenomap-1 and UNL-CPPD are 102.96 GB and 7.73 GB, respectively.

**Fig. 9:**
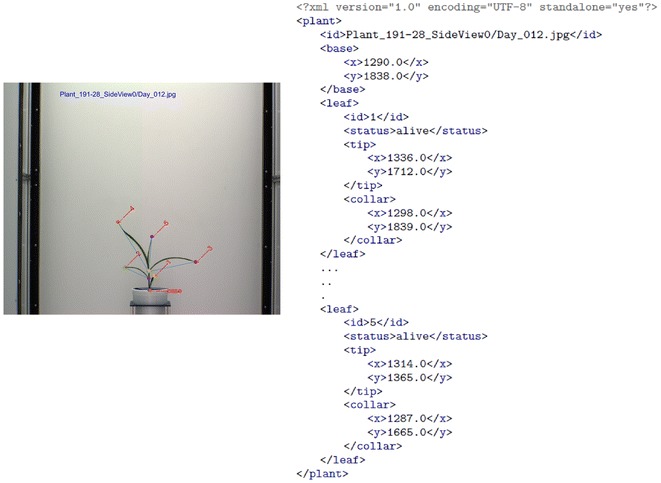
An example of UNL-CPPD ground truth

Figure 9 shows the ground truth of a sample plant from UNL-CPPD. The root element in this XML document is *plant* which contains three child elements, i.e., *id*, *base* and *leaf*.

**Id**: The element *id* is of simple type, i.e., it does not contain any children or attribute. It serves two purposes: (a) when it occurs inside the *plant* element, it refers to the image of the plant of which ground truth information is represented by the XML document; and (b) when it occurs inside a *leaf* element, it represents the leaf number in order of its emergence.

**Base**: The element *base* is of complex type, i.e., it contains children or attributes. It contains 2 children, i.e., *x* and *y*, representing the coordinates of the pixel location of the base.

**Leaf**: The element *leaf* is of a complex type which contains four children elements, i.e., *id*, *status*, *tip* and *collar*. The *leaf* element may appear multiple times in *plant* depending on the number of leaves the plant currently has or had in its life cycle. The child *id* as mentioned before contains the leaf emergence order. *status* element represents the status of the leaf (alive, dead or missing). The status *alive* simply means that the leaf is alive and visible in the image at the given location. The *dead* status means that the leaf appears to be dead in the image mainly due to the separation from the plant stem. The *missing* status means that the leaf is not visible in the image because the leaf might either be dead and no more visible or might be occluded because of the camera angle. The *tip* element has children *x* and *y* which represents the coordinates of the pixel location of the leaf tip, similarly the *collar* element represents the coordinates of the pixel location of the leaf-junction.

## Results

### Holistic phenotyping analysis

**Fig. 10:**
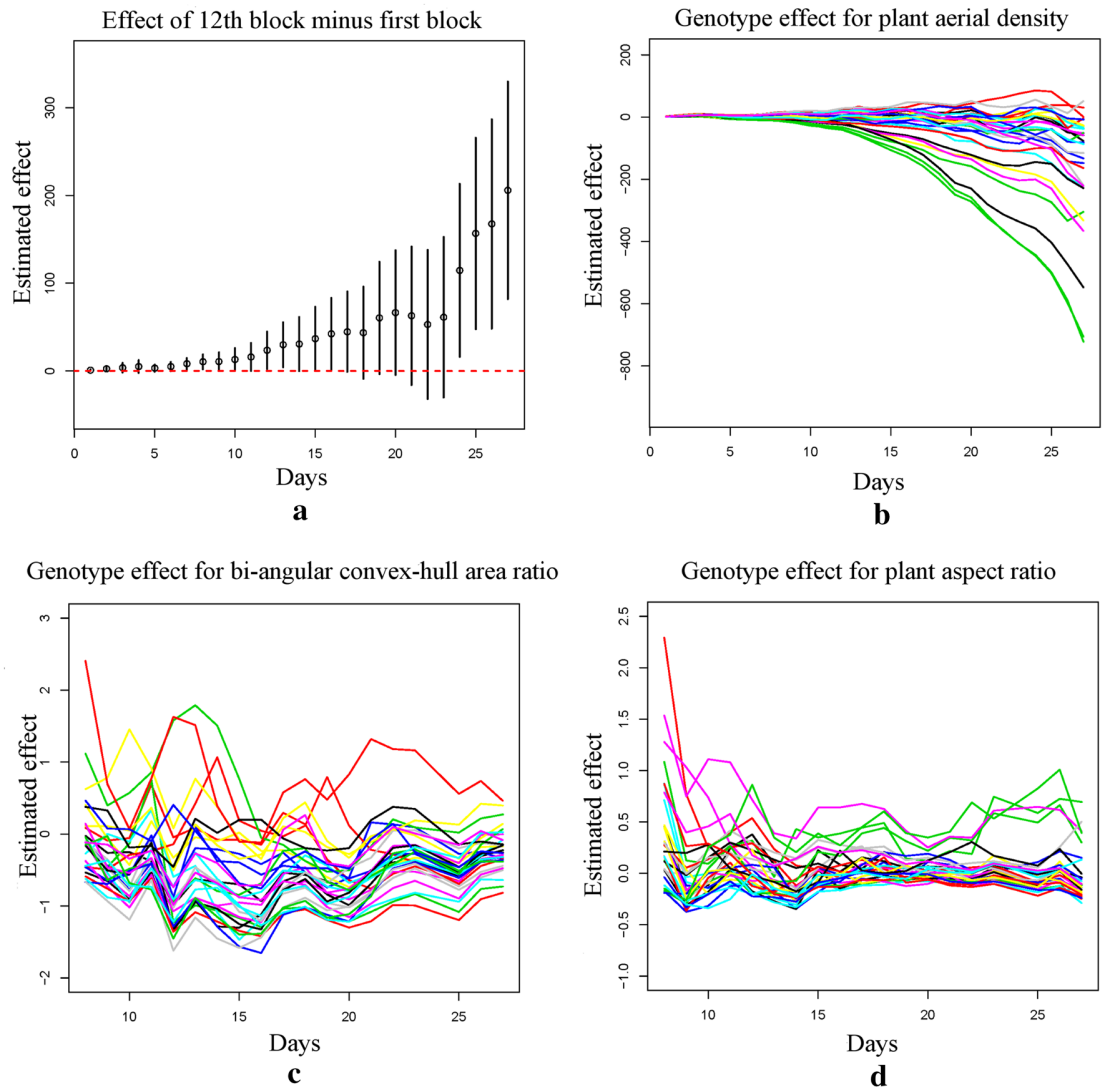
**a** Estimated greenhouse row effect: the differences (denoted by round dots) between the 12th block (in the 6th row, center of greenhouse) and the first block (in the first row) over time, with 95% confidence intervals (denoted by the vertical bars); Genotype effect over time after adjusting the greenhouse row effect, treating the first genotype as the benchmark (the 32 genotypes are denoted by different colors) for **b** plant aerial density; **c** bi-angular convex-hull area ratio and **d** plant aspect ratio

We focus our study on the 32 genotypes of maize, and analyzed three holistic phenotypes extracted from the images: plant aerial density, bi-angular convex-hull area ratio and plant aspect ratio. In the greenhouse, each row (represented as matrix columns in Table 4) is about one meter away from its neighboring row, while the pots in the row are right beside each other. Since the rows are further apart than the columns (represented as matrix rows in Table 4), we grouped the first eight columns in each row as a block, and the rest eight columns as another block. In this way, there are 20 blocks with two in each row. Those blocks were used to quantify the greenhouse environment differences. Please see Table 4 for the experimental design used in this study.

We used a linear regression model to analyze the genotype effect and greenhouse row effect on the plant holistic traits. The responses were modeled independently for each day as

16 $$\begin{aligned} y_{h, ij, t} = \mu _{h, t} + \alpha _{h, i, t} + \gamma _{h, \nu (i, j), t} + \epsilon _{h, ij, t}, \end{aligned}$$

where the subscript $h = 1, 2, 3$ denotes the three kinds of responses: plant aerial density, bi-angular convex-hull area ratio and plant aspect ratio. The subscripts *i*, *j* and *t* denote the *i*th block, *j*th plant in this block and day *t*, respectively, and $\nu (i, j)$ stands for the genotype at this pot, which is determined by the experimental design. The parameters $\alpha$ and $\gamma$ denote block effect and genotype effect, respectively. The error term is denoted as $\epsilon _{h, ij, t}$.

For the response plant aerial density, we first studied the block effect. Understanding greenhouse environment impact is important, since it may confound with the genotype effect of interest. Based on our model, the block effect was not significant in the first few days when all the plants were relatively small. However, this environmental effect became stronger as the plants grew. For the last few days of the experiment, the rows in the middle of the greenhouse had significant positive effect on the plant aerial density in contrast to the rows on the edges. This means that besides the genotype difference, the plants in the middle of the greenhouse grew more than those on the two sides. Specifically, the 12th and 13th blocks in the 6th row had the largest effect, while the effects of the 1st and 2nd block in the first row and 18th and 20th blocks in the last two rows were smallest. One explanation for this is that plants in the center rows experienced light competition from surrounding plants and as a result responded by increasing in height relative to edge plants. This phenomenon is well known and regularly observed under controlled environment and in the field.

Figure 10a plots the estimated row difference between the 12th block and the 1st block over time, where the round dot is the estimated effect and the vertical bar gives the corresponding 95% confidence interval. From Fig. 10a, we see that the confidence intervals are higher than zero for the last 3 days, indicating significant positive effect of the 12th block over the 1st block. After adjusting the block effect, the genotype effect was quite significant. This is due to the choice of the 32 genotypes of maize in our study, which exhibit significant biological difference. Figure 10b plots the adjusted genotype effect over time when treating the first genotype as the benchmark to compare. From this graph, we see that the plants exhibit significant genotype differences even after a few days of germination, and those differences increase as the plants grow.

For the responses bi-angular convex-hull area ratio and plant aspect ratio, we conducted similar analysis and found the block effect is not significant for those two responses. This means the greenhouse layout mainly affects the plant aerial density, but it does not have a significant impact on those two shape-based phenotypic traits of plants. The genotype effect for the bi-angular convex-hull area ratio is significant from the Day 11 to Day 16 of the experiment. This ratio index reflects the plant rotation. Our finding suggests that the genotypes significantly affect the plant rotation around the 2nd week of germination. We also find that the genotype effect is significant for the plant aspect ratio. Please see Fig. 10c, d for the detail comparisons between genotypes for those two traits.

**Fig. 11:**
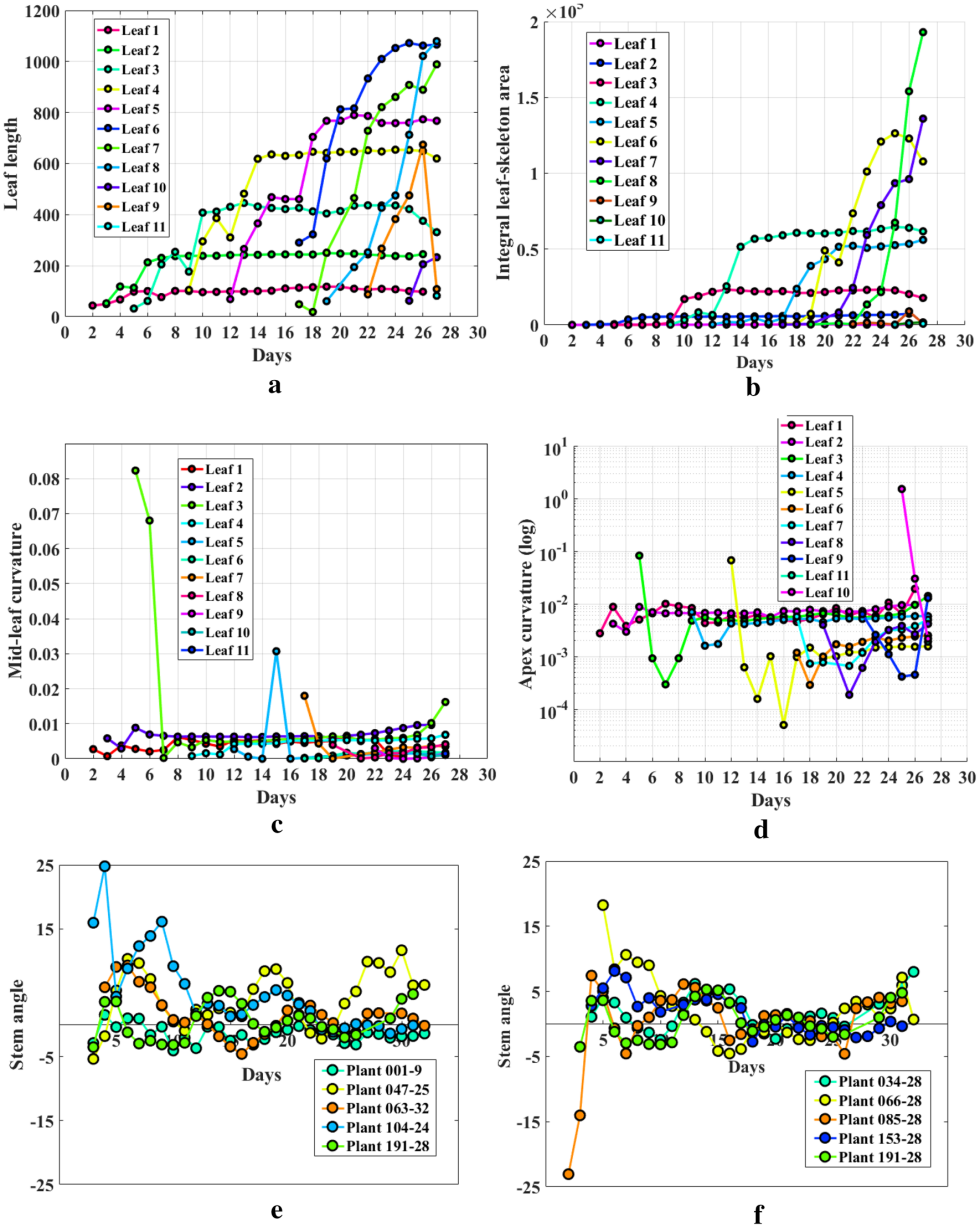
Illustration of temporal variation of component phenotypes: **a** leaf length; **b** integral leaf-skeleton area; **c** mid-leaf curvature; **d** apex-leaf curvature; **e**, **f** stem angle

### Component phenotyping analysis

**Fig. 12:**
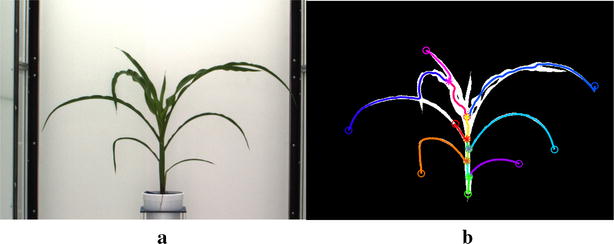
Illustration of leaf detection performance due to leaf crossovers and self-occlusions. **a** Original plant image and **b** detected leaves marked with distinct colors

Experimental analyses are performed on UNL-CPPD to study the temporal variation of the component phenotypes over the vegetative stage life cycle of the maize plants regulated by genetic variations. Here, we manually number the leaves in order of emergence and track them in the images from Day 1 to Day 27.

Figure 11a shows the lengths of each of 11 leaves (shown in different colors) of the plant006-25 starting from the day on which the leaf emerged until Day 27. This figure provides much important information: (a) the day on which a particular leaf emerges; (b) the total number of leaves that are present in the plant on a particular day; (c) the growth pattern of each leaf; and (d) the total number of leaves emerged during vegetative stage life cycle of the plant. For example, it is evident from Fig. 11c that leaf 1 was born on Day 2, while leaf 11 was born on Day 19. The growth rate of leaf 1 is the lowest, while leaf 7 shows significantly high growth rate. On Day 20, the total number of leaves present in the plant is 8.

The temporal variation of integral leaf-skeleton area is shown in Fig. 11b. The figure shows that the integral leaf-skeleton area exhibits similar characteristic feature as that of the leaf length, i.e., the leaves that emerge in the later stage of the life cycle (e.g., leaf 9) has higher value for this component phenotype than the leaves that emerge earlier (e.g., leaf 2). Figure 11c shows the values of mid-leaf curvature for each of 11 leaves for the plant 006-25 emerged in order against increasing days. The variation of apex-leaf curvature of the different leaves are not clearly visible in the linear scale. Thus, we use logarithmic scale to plot the values of apex-leaf curvature for each of 11 leaves against increasing days (see Fig. 11d).

Figure 11e, f show comparisons between inter-genotype and intra-genotype variation of stem angles over time. The values of stem angles in radians (along y-axis) are plotted against the 27 consecutive days (along the x-axis). Figure 11f uses five plants of the same genotype to demonstrate the intra-genotype variation, while Fig. 11e uses five plants of five different genotypes to demonstrate the inter-genotype effect on stem angle. In this study, stem angle is measured from the plants under similar environmental conditions. It is evident from the figures that stem angle is likely to be controlled by genotypic effect. Since, the focus of the paper is to introduce a novel algorithm to compute stem angle as a component phenotype, detailed experimental study to evaluate the genetic influence on stem angle under water-logged or nutriment imbalance conditions, is beyond the scope of this paper.

## Discussion

### Performance evaluation

The plant-level accuracy of algorithm 1 is given by

17 $$\begin{aligned} \text {Plant-level accuracy}=\frac{\sum _{i=1}^n\frac{N_{di}-N_{fi}}{N_{G_i}}}{n}, \end{aligned}$$

where, $N_d$ denotes the number of detected leaves, $N_f$ denotes the number of leaves that are wrongly detected, and $G_i$ denotes the ground truth, i.e., number of leaves present in the plant image $\forall$ *i* = 1,...,*n*, where *n* denotes the total number of images in a plant sequence, i.e., *n* = 27.

The Fig. 12 shows the inaccuracy in leaf detection for an image from UNL-CPPD-II (PlantID: $Plant\_{191}-28^*$, side view 0$^\circ$, Day 30) due to self occlusion and leaf crossover. Table 5 presents the plant-level accuracy corresponding to each plant sequence for both UNL-CPPD-I and UNL-CPPD-II. The average plant-level accuracy for UNL-CPPD-I and UNL-CPPD-II are 92 and 85%, respectively. There are the following three observations. (a) For some plants (e.g., $Plant\_{016}-20^+$), the plant-level accuracy for UNL-CPPD-II is higher than that of UNL-CPPD-I. This is attributed to the fact that these plant sequences contain more images in UNL-CPPD-II compared to its smaller version (UNL-CPPD-I) but none of the additional images has crossovers. (b) In contrast, if most of the additional images of UNL-CPPD-II for a sequence have self-occlusions and leaf crossovers, the accuracy is decreased (e.g., Plant-ID: $Plant\_{063}-32^\dagger$). (c) The plant-level accuracy remains fairly similar for both UNL-CPPD-I and UNL-CPPD-II (e.g., $Plant\_{104}-24^\ddagger$).

### Implementation and run-time details

The algorithms to compute three holistic phenotypes, i.e., (a) bi-angular convex-hull area ratio, (b) plant aspect ratio and (c) plant aerial density, are implemented using OpenCV and C++ on Visual Studio 2010 Express Edition. The original images of the 32 genotypes of the total number of 176 maize plants from the Panicoid Phenomap-1 dataset for two views, i.e., side-view 0$^\circ$ and side-view 90$^\circ$, for 27 days are used to compute the phenotypes that are subsequently analyzed. The time to compute the three holistic phenotypes on 176 $\times$ 27 $\times$ 2 = 9504 images using an Intel(R)Core(TM) i7 processor with 16 GB RAM working at 2.60-GHz using 64 bit Windows 7 operating system are respectively 2.15, 2.23 and 2.05 h. Algorithm 1 is implemented using Matlab R2016a on the same platform. We record the total time taken to execute Algorithm 1 on 13 $\times$ 27 = 351 images (13 plants for one side-view for 27 days) of UNL-CPPD as 3 h 20 min. Thus, the average execution time of a single plant sequence is 15.38 min.

**Table 5:** Performance summary of algorithm 1 on UNL-CPPD dataset (Naming convention for plant sequence is: Plant_ID-Genotype ID [1])

Plant sequence	Dataset	No. leaves	Detected leaves	False leaves	Accuracy
$Plant\_{001}-9$	CPPD-I	116	93	1	0.79
	CPPD-II	168	157	5	0.83
$Plant\_{006}-25$	CPPD-I	138	136	0	0.98
	CPPD-II	205	188	5	0.91
$Plant\_{008}-19$	CPPD-I	142	140	0	0.98
	CPPD-II	210	200	9	0.86
$Plant\_{016}-20^+$	CPPD-I	103	86	0	0.83
	CPPD-II	141	129	0	0.88
$Plant\_{023}-1$	CPPD-I	113	101	0	0.89
	CPPD-II	154	135	8	0.83
$Plant\_{045}-1$	CPPD-I	122	120	3	0.96
	CPPD-II	177	170	6	0.93
$Plant\_{047}-25$	CPPD-I	148	142	2	0.94
	CPPD-II	212	196	5	0.88
$Plant\_{063}-32^\dagger$	CPPD-I	149	138	0	0.93
	CPPD-II	214	174	18	0.72
$Plant\_{070}-11$	CPPD-I	125	111	0	0.89
	CPPD-II	177	148	5	0.83
$Plant\_{071}-8$	CPPD-I	141	131	0	0.93
	CPPD-II	199	163	7	0.77
$Plant\_{076}-24$	CPPD-I	135	126	2	0.92
	CPPD-II	191	152	2	0.78
$Plant\_{104}-24^\ddagger$	CPPD-I	144	140	0	0.97
	CPPD-II	186	185	0	0.96
$Plant\_{191}-28$*	CPPD-I	137	111	0	0.96
	CPPD-II	178	151	7	0.81
Average	CPPD-I	132	123	< 1	0.92
	CPPD-II	186	165	$\approx$ 6	0.85

* Plant sequence used to demonstrate inaccuracy in leaf detection due to self-occlusion and leaf crossover

^+^Plant-level accuracy for UNL-CPPD-II is higher than that of UNL-CPPD-I

^†^Plant-level accuracy for UNL-CPPD-II is lower than that of UNL-CPPD-I

^‡^Plant-level accuracy remains fairly similar for both UNL-CPPD-I and UNL-CPPD-II

## Conclusion

We classify image-based plant phenotypes into two categories: holistic and component. Holistic phenotypes are computed by considering the whole plant as a single object, whereas component phenotypes represent the traits of the individual components of the plants, e.g., stem and leaves. Experimental analysis performed on our publicly available dataset called Panicoid Phenomap-1 demonstrate the genetic regulation of the three holistic phenotypes, namely, bi-angular convex-hull area ratio, plant aspect ratio and plant aerial density, in maize. Bi-angular convex-hull area ratio is a measure of plant rotation due to shade avoidance, and provides information on phyllotaxy, i.e., the arrangement of leaves around a stem. plant aspect ratio and plant aerial density provide information on canopy architecture and plant biomass, respectively.

The vigor of a maize plant is best interpreted by the emergence timing, total number of leaves present at any development stage and the growth of individual leaves. To compute these phenotypes based on imaging techniques, it is essential to reliably detect the individual leaves of the plants. Thus, the paper introduces a novel algorithm to detect and count the total number of leaves of a maize plant by analyzing 2D visible light image sequences using a graph based approach. We have also presented algorithms to compute six component phenotypes, namely, leaf length, junction-tip distance, leaf curvature (two types: mid-leaf curvature and apex-curvature), junction-tip angle, integral leaf-skeleton area and stem angle. While leaf length and junction-tip distance contribute to the study of growth monitoring of the plants, leaf curvature helps in the measurement of leaf toughness. Stem angle (a measure for the displacement of the stem away from the vertical axis) is a determining factor of plant’s susceptibility to lodging, i.e., bending of the stem. Lodging is primarily caused by the water-logged soil conditions and nutrient imbalances and deficiencies [31].

The proposed method provides an extensive study on holistic and component phenotypes in maize with significance in plant science. It automatically detects each leaf of a maize plant to derive a number of new component phenotypes compared to the recent state-of-the-art methods (e.g., [9, 13]) from image sequences for temporal plant phenotyping analysis. To evaluate the performance of our algorithm and stimulate research in this area, we introduce a benchmark dataset, i.e., UNL-CPPD. The dataset consists of a set of maize plants along with the list of leaves and their end coordinates manually determined to be ground truth. Experimental analyses are performed on UNL-CPPD to demonstrate the temporal variation of the component phenotypes in maize regulated by different genotypes. The proposed plant architecture determination algorithm does not take into consideration self-occlusions due to leaf crossovers. Therefore, future work will consider to advance the algorithm to deal with self-occlusions. In addition, an automatic leaf tracking in the presence of self-occlusion and view variations will also be considered in the future work.
